# Neurocysticercosis in a Non-Endemic Region: A Retrospective Cohort Study at Sheikh Khalifa Medical City (SKMC), Abu Dhabi, UAE

**DOI:** 10.7759/cureus.85082

**Published:** 2025-05-30

**Authors:** Mohammed El-Lahawi, Sara M Moustafa, Shahad ElHag, Mohamed M Abd Elhamid, Ahmad Nizam, Fazil T Manzil, Lamya Turkawi, Salah Abdelrahman, Mustafa Shakra

**Affiliations:** 1 Neurology Department, Sheikh Khalifa Medical City, Abu Dhabi, ARE; 2 Internal Medicine Department, Sheikh Khalifa Medical City, Abu Dhabi, ARE; 3 Gastroenterology Department, Cleveland Clinic Abu Dhabi, Abu Dhabi, ARE; 4 Education Department, Sheikh Khalifa Medical City, Abu Dhabi, ARE

**Keywords:** cystic brain lesions, cysticercosis, neglected tropical diseases, neurocysticercosis, non-endemic region, taenia solium, uae

## Abstract

Introduction

Neurocysticercosis (NCC) is the most common parasitic disease of the nervous system and a leading cause of acquired epilepsy. To our knowledge, this is the first study to describe the clinical presentation, radiological manifestations, short-term outcomes, and treatment of patients diagnosed with neurocysticercosis (NCC) at a tertiary care center in the United Arab Emirates (UAE), conducted at Sheikh Khalifa Medical City (SKMC), Abu Dhabi. This study aims to describe the demographics, presenting symptoms, neuroimaging patterns, and treatment responses of patients diagnosed with NCC in a non-endemic setting over a 10-year period. This study also aims to improve healthcare provider awareness of neurocysticercosis in a non-endemic region.

Methods

A retrospective review was conducted using the charts from the electronic medical records of adult patients (>16 years of age) with confirmed diagnosis of NCC who were admitted to SKMC between January 1, 2012 and December 31, 2021. Demographic data, symptoms at presentation, neuroimaging findings and treatment were retrieved from the medical records of these patients.

Results

During the period between 2012 and 2021, 17 patients who were eventually diagnosed with NCC were admitted to the medical ward through the emergency department at SKMC. Most patients (n=14, 82.4%) were aged between 16 and 37 years. Only one patient was female (n=1, 5.9%), yielding a male-to-female ratio of 16:1. All patients were from Southeast Asia, with the majority (n=12, 70.6%) originating from India. The main presenting symptom was generalized tonic-clonic seizures, observed in 14 (82.4%) patients. These patients were started on antiepileptic drugs (AEDs) after the diagnosis of NCC was confirmed. They were advised to follow up with their primary care physician for ongoing management and continuation of care. Other presenting symptoms were equally seen, including hemiparesis (n=1, 5.9%), headaches (n=1, 5.9%), and psychiatric manifestations (n=1, 5.9%). All patients (100%) had a Computed Tomography (CT) head done on admission, and all of them had cysts typical of NCC on their imaging studies. Eleven patients (n=11, 64.7%) had one cyst only, while three (n=3, 17.6%) had more than four cysts. The most common location was the frontal lobe (n=7, 41.2%), followed by the parietal lobe (n=6, 35.3%). In terms of antiparasitic initiation, only one patient was not put on any antiparasitic therapy since he had a calcified lesion. Nine patients (53%) received prednisolone at 1mg/kg/day, tapered over 10 days.

Conclusion

NCC is a common cause of seizures and should be considered in the differential diagnosis of patients presenting with seizures, particularly in expatriates from endemic regions. Diagnosis should follow standardized criteria such as Del Brutto’s, and albendazole remains first-line therapy unless lesions are calcified. While NCC is not directly contagious, its transmission through ingestion of *Taenia solium* eggs underscores the importance of hand hygiene practices and preventive health education. This study highlights the need for greater clinical awareness and further research to support public health planning in non-endemic settings.

## Introduction

Neurocysticercosis (NCC) represents the leading parasitic condition affecting the human brain [1]. NCC occurs as a result of ingestion of Taenia solium eggs via fecal-oral contamination from a taenia carrier or due to auto-infection by the fecal-oral route in individuals carrying the adult intestinal tapeworm. The ingestion of undercooked pork leads to transmission of Taenia solium to humans but doesn't cause NCC [1]. Following ingestion of *Taenia solium* eggs, *Taenia solium* larvae migrate and become encysted, typically in the muscle tissue of the host. When *Taenia solium* cysticerci develops in the human brain, the condition is defined as NCC [1].

According to the World Health Organization (WHO), *Taenia solium* is one of the major contributors to mortality among foodborne illnesses, affecting an estimated 2.5 to 8.3 million people worldwide with neurocysticercosis [2]. Approximately 80% of global cysticercosis cases are concentrated in developing regions, particularly in Latin America, sub-Saharan Africa, and across South and Southeast Asia. NCC is recognized by the WHO as a neglected tropical disease. Despite this, it remains the leading cause of preventable epilepsy and neurological impairment in adults globally [3]. Other clinical manifestations vary including focal neurological deficits and headache. Furthermore, NCC creates a tremendous economic burden. Over the past decade, NCC has been estimated to cost the U.S. healthcare system more than one billion dollars [4].

Neurocysticercosis can also occur in high-income, non-endemic countries, primarily among immigrants originating from endemic regions. The United Arab Emirates (UAE), a prosperous Gulf nation, hosts a large expatriate population. Many of these individuals come from countries such as India, Bangladesh, Nepal, the Philippines, and Ethiopia - regions where *Taenia solium* taeniasis and similar parasitic infections remain prevalent. This has led to the emergence of many tropical and zoonotic infections that are rarely seen in this part of the world. Although the UAE is not an endemic area for *Taenia solium* transmission or NCC, imported cases continue to emerge due to population mobility and global migration. To date, there are no epidemiological or descriptive studies of the prevalence, manifestations, or the burden of NCC in the UAE.

However, a recently published study conducted in Kuwait in 2021, studying the prevalence of NCC in the country over the past 10 years, reported that out of 970 high-risk patients screened, 150 (15%) of them had positive serology (enzyme-linked immunoelectrotransfer blot assay or EITB) for cysticercosis. Surprisingly, of the total population screened, 52 (5.4%) Kuwaitis were found to have cysticercosis [5]. Those significant findings in a neighboring country highlight the need for similar studies to illustrate the prevalence, burden, and characteristics of this disease in the UAE. Shedding light on this disease will help improve healthcare providers’ understanding of the presentations, diagnostic procedures and treatment, which will result in a better approach reducing mortality, morbidity and financial costs. This study aims to address that gap by describing the demographics, presenting symptoms, neuroimaging patterns, and treatment responses of NCC patients admitted to a tertiary hospital in the United Arab Emirates over a 10-year period.

## Materials and methods

Study design

We conducted a retrospective study of adult patients diagnosed with NCC at Sheikh Khalifa Medical City (SKMC), a 500-bed tertiary care teaching hospital located in Abu Dhabi, United Arab Emirates. The study was conducted by reviewing the medical records charts of patients in the period from January 1, 2012 to December 31, 2021. We selected the year 2012 as the starting year due to limitations in retrieving electronic medical records prior to that point. Ethical approval was obtained from the Institutional Review Board of SKMC (IRB #777), and the requirement for informed consent was waived due to the retrospective and anonymized nature of the data.

Study population

Patients aged 16 years and above with a confirmed diagnosis of NCC - defined based on clinical or epidemiologic exposure criteria and characteristic neuroimaging findings on Computed Tomography (CT) or Magnetic Resonance Imaging (MRI) consistent with Del Brutto's criteria - who were treated at SKMC between January 1, 2012 and December 31, 2021, were included [6]. Pediatric patients aged 16 years or younger were excluded from the study. A total of 17 patients met the inclusion criteria and were included in the final analysis. No formal sample size calculation was performed due to the retrospective and descriptive nature of the study. All patients meeting the inclusion criteria during the 10-year study period were included.

Study variables and outcomes

Data were abstracted from the electronic medical records using a standardized data collection tool and included demographic variables (age, gender, nationality), clinical presentation (seizures, headache, hemiparesis, psychiatric symptoms), past medical history, imaging modality used (CT, MRI), neuroimaging findings (number, stage and location of lesions), and treatments received (antiparasitic therapy, corticosteroids). Primary outcomes included presenting symptoms, imaging features, and management strategies. Data abstraction was performed by a single reviewer using a standardized data collection form. All extracted data were subsequently cross-verified for consistency and completeness to minimize potential bias. Long-term follow-up was not available; however, all patients were seen at least two to three times in outpatient follow-up. No seizure recurrence or new symptoms were reported during this short-term period, and antiepileptic therapy was continued per neurologist recommendation.

Statistical analysis

Data were analyzed using IBM SPSS Statistics software Version XX (IBM Corp., Armonk, NY, USA). Descriptive statistics were used to summarize demographic characteristics, clinical presentations, imaging findings, and treatments. Categorical variables are presented as frequencies and percentages. Given the small sample size, no inferential statistical tests were performed.

## Results

Patients’ demographics

Between 2012 and 2021, 17 patients were admitted and diagnosed with neurocysticercosis (Table 1). Most of the patients were between 16 and 30 years of age (n=9, 52.9%). All patients were expatriates from Southeast Asia with the majority being from India (n=12, 70.6%), followed by Nepal (n=3, 17.6%) and one each from Bangladesh and Yemen. Only one patient had a previous diagnosis of neurocysticercosis in the past. Only one patient was female (5.9%), yielding a male-to-female ratio of 16:1. This may reflect the regional workforce demographics in the UAE, where the expatriate labor population is predominantly male. However, given the small sample size, this distribution should be interpreted with caution.

**Table 1:** Patients demographics (n=17)

Demographic Category	Subcategory	Data (%)
Gender	Male	16 (94.1%)
	Female	1 (5.9%)
Ethnicity	India	12 (70.6%)
	Nepal	3 (17.6%)
	Bangladesh	1 (5.9%)
	Yemen	1 (5.9%)
Age Group	16–30 years	9 (52.9%)
	30-40 years	6 (35.3%)
	40+ years	2 (11.8%)

A case-level summary of each patient’s demographics, clinical presentation, neuroimaging characteristics, and treatment is provided in Table 2.

**Table 2:** Individual Demographic, Clinical, and Treatment Characteristics of Patients with Neurocysticercosis (n = 17)

Patient #	Age Group	Country	Symptoms	Number of Cysts	Cyst Location	Lesions stage	Antiparasitic	Steroid	
1	16-30	India	Seizures	1	Frontal lobe	calcified	No	No	
2	30-40	India	Seizures	1	Parietal lobe	granular nodular	Yes	No	
3	16-30	India	Occipital headache, left hand and left leg numbness, focal seizures	1	Frontal lobe	mixed colloidal vesicular and granular nodular	Yes	Yes	
4	30-40	Nepal	Seizures	2	Frontal lobe and Occipital lobe	mixed granular nodular and nodular calcified	Yes	No	
5	16-30	India	Seizures	3	Right parieto-occipital region, left insula, left occipital lobe	mixed colloidal vesicular, granular nodular and nodular calcified	Yes	Yes	
6	16-30	Yemen	Seizures	1	Temporal lobe	mixed colloidal vesicular and granular nodular	Yes	Yes	
7	30-40	India	Seizures	4+	Grey white matter junctions, left thalamus, Parietal lobe	vesicular and colloidal vesicular	Yes	Yes	
8	16-30	Nepal	Seizures	1	Occipital lobe	colloidal vesicular	Yes	No	
9	16-30	India	Agitation, Depressive symptoms, insomnia, decreased appetite	2	Parietal lobe	mixed granular nodular and nodular calcified	Yes	No	
10	30-40	India	Seizures	4+	Temporal lobe	colloidal vesicular	Yes	Yes	
11	30-40	Bangladesh	Seizures	1	Frontal lobe	colloidal vesicular	Yes	Yes	
12	16-30	Nepal	Seizures	1	Parietal lobe	mixed granular nodular and nodular calcified	Yes	No	
13	16-30	India	Seizures	1	Frontal lobe	mixed colloidal vesicular and nodular calcified	Yes	No	
14	30-40	India	Seizures	1	Frontal lobe	granular nodular	Yes	No	
15	40+	India	Right-sided hemiparesis	1	Left Posterior limb internal capsule	colloidal vesicular	Yes	Yes	
16	16-30	India	Seizures	1	Parietal lobe	colloidal vesicular	Yes	Yes	
17	40+	India	Seizures	4+	Frontal lobe, temporo-occipital lobe, parietal lobe, cerebellum	mixed colloidal vesicular and nodular calcified	Yes	Yes	

Clinical presentation

**Figure 1:**
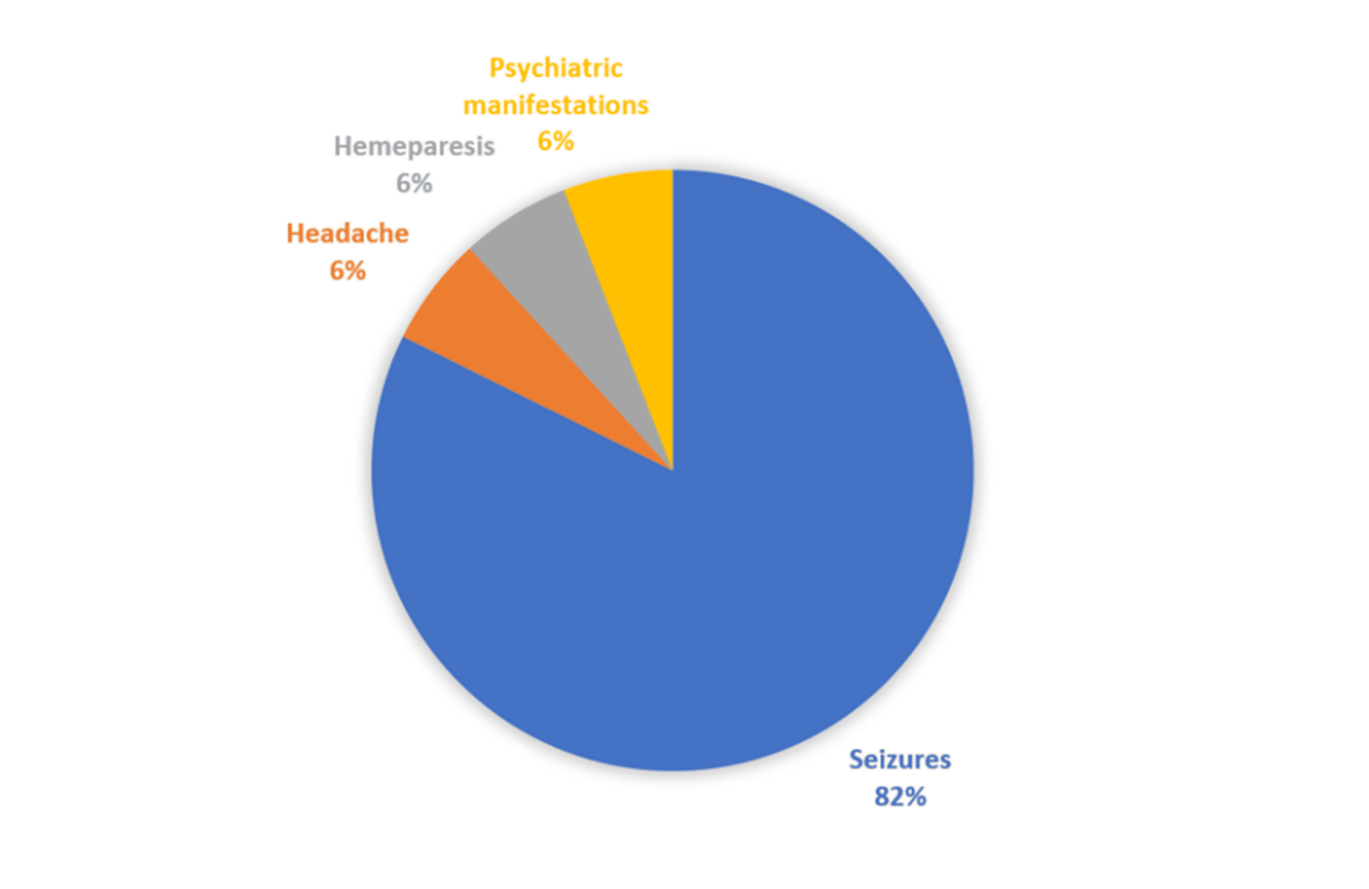
Clinical Presentations

Seizures were the most frequent presenting symptom (n=14, 82.4%). Other symptoms included hemiparesis (n=1, 5.9%), headaches (n=1, 5.9%), and psychiatric manifestations such as agitation, depression, or insomnia (n=1, 5.9%) as shown in Figure 1.

Management and approach to patients

**Figure 2:**
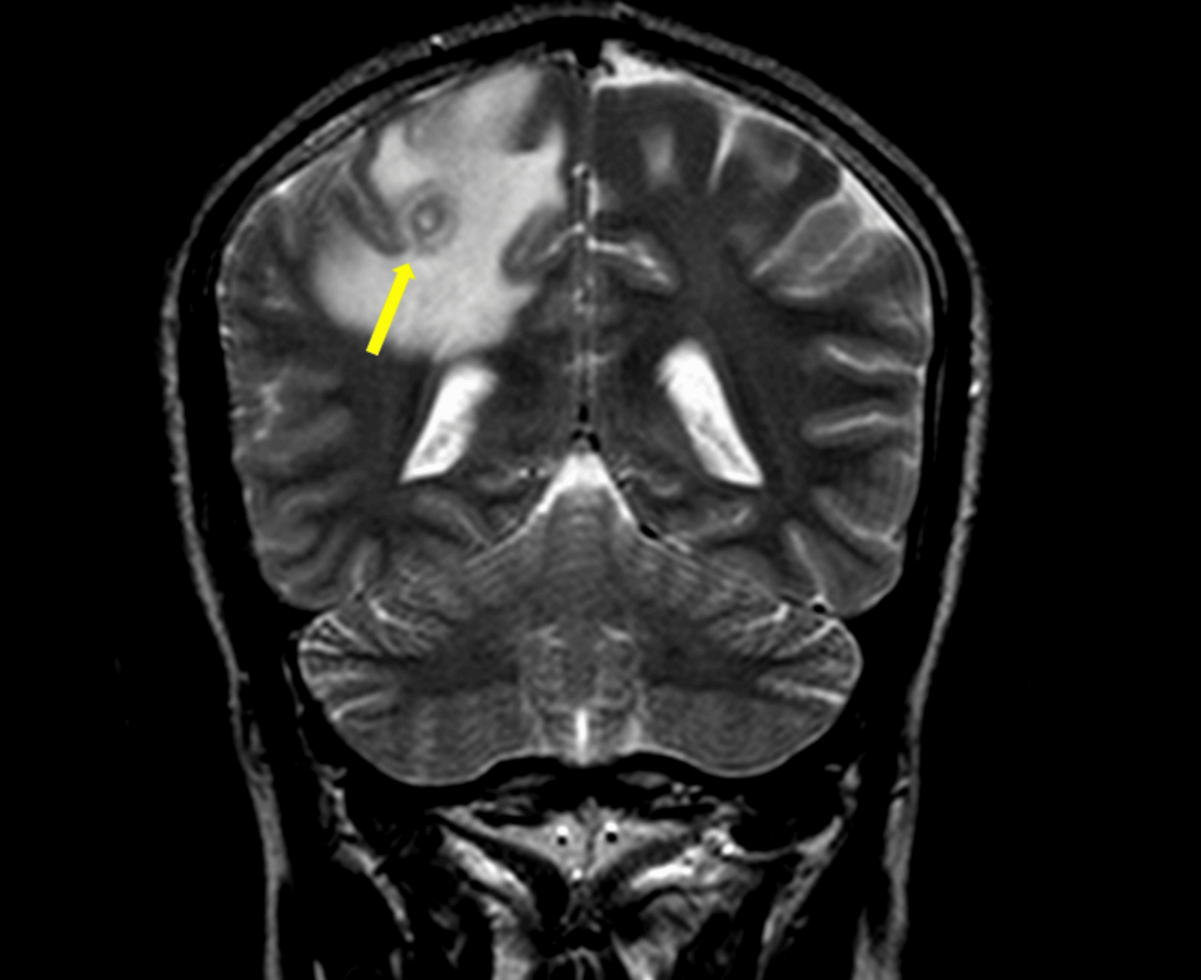
Coronal Brain MRI showing subcortical grapelike ring-enhancing lesions in the right parietal lobe with surrounding right fronto-parietal lobe vasogenic oedema. These features are consistent with the colloidal vesicular stage of NCC.

All patients underwent non-contrast, contrast-enhanced brain CT and brain MRI scans. Imaging revealed lesions typical of neurocysticercosis, with solitary cysts observed in 64.7% (n = 11) of patients and more than four cysts in 17.6% (n = 3). Based on radiology reports, lesions were staged according to established classifications: vesicular, colloidal vesicular, granular nodular, or nodular calcified (Table 2). Perilesional edema was noted in 12 cases (70.6%), particularly those with colloidal lesions. One representative MRI image (Figure 2) shows a ring-enhancing lesion in the right parietal subcortical region with associated fronto-parietal lobe vasogenic edema, consistent with the colloidal vesicular stage of NCC. No alternative diagnoses were proposed in the original imaging interpretations. Differential diagnoses for isolated calcified lesions - such as tuberculomas, toxoplasmosis, and gliomas - were considered and ruled out based on clinical context, lack of systemic features, serological testing, and characteristic imaging findings.

**Figure 3:**
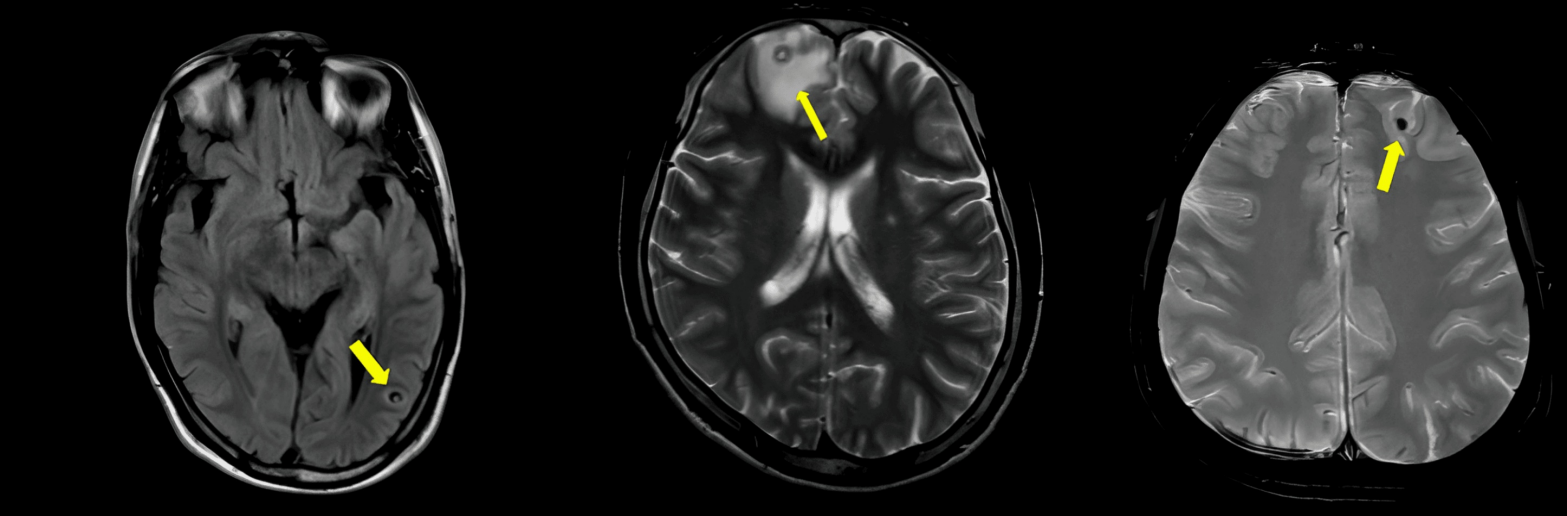
Brain MRI images of NCC in different stages: Vesicular stage (Left), Colloidal vesicular stage (Middle), and Calcified stage (Right).

Patients presented with NCC at various stages, with many exhibiting mixed-stage lesions. The most common predominant stage was the colloidal vesicular stage, observed in 10 patients (58.8%) (Figure 3).

**Figure 4:**
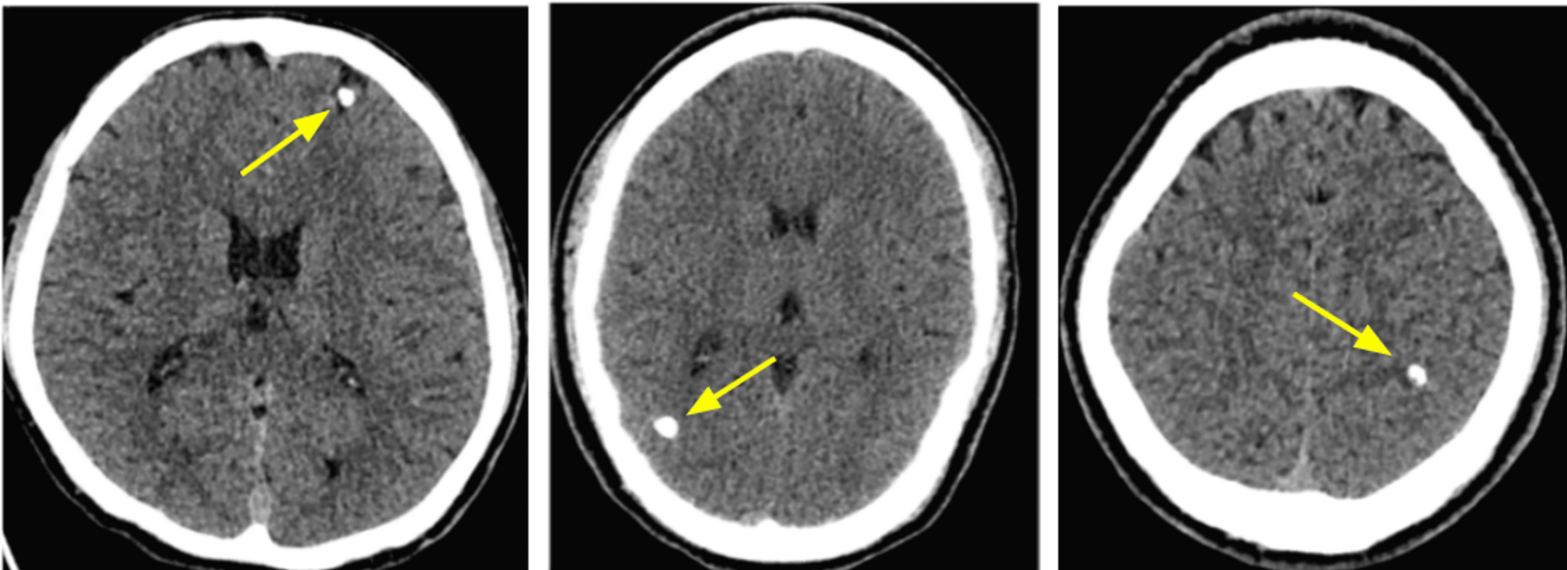
Imaging findings in patients with parenchymal brain cysticercosis, including Frontal (Left), Occipital (Middle), and Parietal lobes (Right).

The lesions were most commonly located in the frontal lobe (n = 7, 41.2%) and parietal lobe (n = 6, 35.3%). Other locations included the occipital lobe, cerebellum, internal capsule, thalamus and grey-white matter junction (Figure 4).

**Figure 5:**
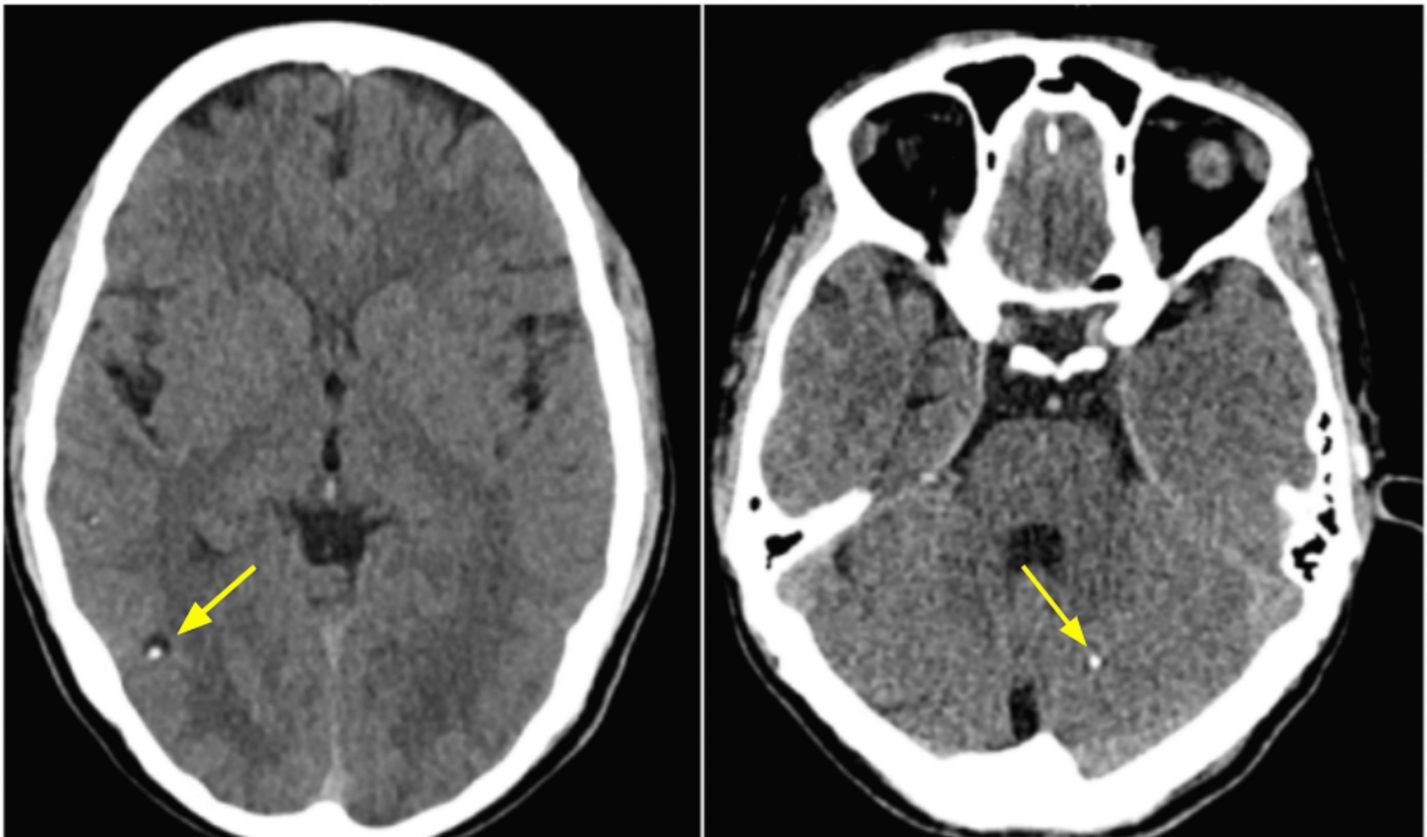
Brain CT imaging findings in a patient with multiple parenchymal neurocysticercosis, showing a vesicular cyst in the right posterior lateral parietal lobe (left), measuring up to 12 mm, and a calcified cyst in the left cerebellum (right).

Some patients presented with multiple cysts (n=6, 35.2%), involving different brain regions and, in some cases, representing mixed evolutionary stages of neurocysticercosis (Figure 5).

None of the patients underwent muscular radiographs for the detection of soft tissue calcifications. All patients received antiparasitic therapy except one patient who had a calcified lesion, and nine patients (53%) received prednisolone at 1mg/kg/day, tapered over 10 days.

## Discussion

The increasing number of NCC patients in developed and non-endemic regions, including the Middle East, can be attributed to global migration and the greater availability of neuroimaging in emergency departments. This trend poses a growing public health challenge, primarily due to a lack of awareness and familiarity with NCC among healthcare providers in non-endemic settings, which can delay diagnosis and treatment. It is also possible that underdiagnosis or underreporting may be occurring in other centers in the UAE due to limited access to serological testing and unfamiliarity with the condition. While NCC itself is not contagious, a small proportion of patients may have concurrent *Taenia solium* taeniasis and can pose a transmission risk to close contacts through fecal-oral contamination. Studies estimate that approximately 5-10% of NCC patients may carry the adult tapeworm [3]. Additionally, individuals from taeniasis-endemic regions may carry the adult *Taenia solium* tapeworm, posing a potential risk for local transmission through fecal-oral contamination [5]. This underscores the importance of considering targeted screening in high-risk populations, particularly in non-endemic regions where clinical awareness may be limited. NCC can mimic several other neurological conditions, including CNS tuberculosis, brain abscesses, gliomas, and metastases, which further complicates clinical evaluation. To the best of our knowledge, this is the first study to examine the clinical features, neuroimaging characteristics, and short-term outcomes of patients diagnosed with NCC in the United Arab Emirates.

In our study, we observed that most of the patients were of a young age, between 16 and 37 years of age with a male predominance. Similar findings have been documented in studies from India and Peru, where the average age of patients diagnosed with NCC was approximately 27 years [7-8]. In contrast, research from countries that are less endemic - such as China, Indonesia, Spain, and the United States - has reported a higher mean patient age, typically between 37 and 40 years [9-12].

All cases were expatriates with the majority being from India, followed by Nepal which are taeniasis-endemic countries. A recently published large case series of NCC patients from Kuwait has also shown that the majority of expatriates originated from the same countries [5].

The most common presenting symptom in our study was generalized tonic-clonic seizures. Other presenting symptoms were focal neurological deficit (including hemiparesis), headaches, and psychiatric manifestations. Our findings align with previous studies in which generalized seizures were also frequently reported among patients with NCC [5,13].

Cysticercal lesions were identified on brain CT or MRI in all 17 patients, with solitary lesions more common than multiple ones. In addition to the clinical presentation and epidemiological exposure criteria, these imaging findings contributed to the diagnosis, in accordance with Del Brutto's diagnostic criteria. Similar results were reported from various previous studies [14-16].

None of the cases in our study had extraparenchymal lesions. A recent study by Berto and Coyle, a large case series from New York City, found that parenchymal neurocysticercosis was present in the majority of patients, while subarachnoid involvement was less common [9]. Current literature generally finds parenchymal NCC to be the predominant form, with extraparenchymal involvement - such as subarachnoid cysticercosis - occurring less commonly [17].

In our study, we documented the anatomical region of cystic lesions by neuroimaging, and we found that the frontal lobe and parietal lobe were more commonly involved. The remaining patients had involvement of either multiple lobes or isolated involvement of the temporal lobe, occipital lobe, cerebellum, or the grey-white matter junction and internal capsule. The stages of neurocysticercosis reflect the evolution of the parasite within the central nervous system and follow well-defined radiological stages - vesicular, colloidal vesicular, granular nodular, and nodular calcified [2]. In our cohort, the colloidal vesicular stage was the most commonly observed (58.8%) and was often associated with perilesional edema. Several patients exhibited mixed-stage lesions, reflecting varied disease progression. Recognition of these stages, along with clinical presentation and epidemiological exposure criteria, helped guide the diagnosis of these patients.

The introduction of two cysticidal drugs (praziquantel and albendazole) has drastically changed the prognosis of most patients with neurocysticercosis. Albendazole has been superior to praziquantel in trials comparing the efficacy of these drugs [18-19]. However, it is important to note that some forms of neurocysticercosis should not be treated with cysticidal drugs, including patients with cysticercotic encephalitis, as it exacerbates the intracranial hypertension, and also patients with calcifications as calcified lesions represent sequelae of previous infections [20]. In our study, all patients were started on Albendazole except one patient who had a calcified lesion. Sometimes, substantial oedema can occur around the cystic lesions, and steroids can help in the acute management of symptoms secondary to moderate or severe perilesional oedema developing around one or more cysts [2]. Nine patients (53%) received prednisolone at 1mg/kg/day, tapered over 10 days. The hospital stay ranged from three to four days for most patients. None of the patients required readmission, and no recurrence of symptoms was documented during follow-up. Patients were typically seen two to three times in outpatient neurology clinics before being referred back to their primary care providers for ongoing management. Follow-up neuroimaging should be performed every six months following completion of antiparasitic therapy until radiographic resolution. For patients with persistent lesions on follow-up imaging, a repeat course of antiparasitic therapy is recommended [21].

Limitations

This study has several limitations. First, it was retrospective in nature, which carries inherent risks of selection bias, missing data, and limited control over diagnostic workups. Although SKMC serves as a referral center for complex cases from other hospitals, the small sample size and single-center design still limit the generalizability of our findings. While neuroimaging findings and clinical features were described, not all patients had standardized serological testing or long-term follow-up imaging. Most patients were followed for two to three outpatient visits after discharge, but were subsequently advised to continue long-term care with their primary care physicians, which limited access to extended outcome data. Despite these limitations, the study provides valuable insight into the clinical presentation and management of NCC in a non-endemic setting.

## Conclusions

This study presents the first UAE-based review of neurocysticercosis, highlighting the clinical characteristics, neuroimaging features, and short-term outcomes of patients diagnosed between 2012 and 2021 at a tertiary center. All NCC cases were expatriates originating from taeniasis-endemic countries. Therefore, NCC should always be considered in patients from endemic areas presenting with seizures, headaches, neuropsychiatric symptoms, or compatible lesions on brain imaging. Diagnosis should be guided by well-established diagnostic criteria such as Del Brutto’s criteria, which incorporate imaging, clinical, and epidemiological features to support diagnostic accuracy. Albendazole remains the first-line antiparasitic agent unless lesions are calcified, in which case antiparasitic therapy may not be indicated. Raising awareness among clinicians is essential to improve recognition, guide appropriate treatment decisions, and avoid unnecessary diagnostic interventions in non-endemic settings. Although NCC is not transmitted directly from person to person, it occurs through ingestion of *Taenia solium* eggs via fecal-oral contamination, highlighting the importance of proper hand hygiene practices. This study, while limited by sample size and scope, highlights the need for broader epidemiological research and may help inform public health initiatives focused on NCC recognition in non-endemic settings.
